# Presence of *Mycoplasma fermentans *in the bloodstream of Mexican patients with rheumatoid arthritis and IgM and IgG antibodies against whole microorganism

**DOI:** 10.1186/1471-2474-10-97

**Published:** 2009-08-03

**Authors:** Constantino Gil, Antonio Rivera, David Bañuelos, Salvador Salinas, Ethel García-Latorre, Lilia Cedillo

**Affiliations:** 1 Centro de Investigaciones en Ciencias Microbiológicas, Instituto de Ciencias, Benemérita Universidad Autónoma de Puebla, Edificio 103J, Ciudad Universitaria, Puebla, Pue, México; 2 Servicio de Reumatología, Centro Médico Manuel Ávila Camacho, Instituto Mexicano del Seguro Social, 2 Norte y 18 Oriente, Puebla, Pue, México; 3 Escuela Nacional de Ciencias Biológicas, Instituto Politécnico Nacional, Prolongación de Plan de Ayala y Carpio, México

## Abstract

**Background:**

Increasing evidence incriminates bacteria, especially *Mycoplasma fermentans*, as possible arthritogenic agents in humans. The purpose of this study was to investigate *M. fermentans *in the bloodstream of patients with rheumatoid arthritis.

**Methods:**

Two hundred and nineteen blood samples from patients with rheumatoid arthritis, systemic lupus erythematosus, antiphospholipid syndrome, and healthy individuals were screened by bacterial culture and direct PCR in order to detect mycoplasmas; IgM and IgG against *M. fermentans *PG18 were also detected by ELISA and Immunoblotting assays in patients with rheumatoid arthritis and healthy individuals.

**Results:**

Blood samples from patients with antiphospholipid syndrome and healthy individuals were negative for mycoplasma by culture or direct PCR. In blood samples from patients with systemic lupus erythematosus were detected by direct PCR *M. fermentans *in 2/50 (2%), *M. hominis *in 2/50 (2%) and *U. urealyticum *in 1/50 (0.5%). In patients with RA *M. fermentans *was detected by culture in 13/87 blood samples and in 13/87 by direct PCR, however, there was only concordance between culture and direct PCR in six samples, so *M. fermentans *was detected in 20/87(23%) of the blood samples from patients with RA by either culture or PCR. Antibody-specific ELISA assay to *M. fermentans *PG18 was done, IgM was detected in sera from 40/87 patients with RA and in sera of 7/67 control individuals, IgG was detected in sera from 48/87 RA patients and in sera from 7/67 healthy individuals. Antibody-specific immunoblotting to *M. fermentans *PG18 showed IgM in sera from 35/87 patients with RA and in sera from 4/67 healthy individuals, IgG was detected in sera from 34/87 patients and in sera from 5/67 healthy individuals.

**Conclusion:**

Our findings show that only *M. fermentans *produce bacteremia in a high percentage of patients with RA. This finding is similar to those reported in the literature. IgM and IgG against *M. fermentans *PG18 were more frequent in patients with RA than healthy individuals.

## Background

Rheumatoid arthritis (RA) is a chronic inflammatory disease, which results from a complex interplay of factors both at the systemic level and at the site of inflammation [1].

Rheumatoid arthritis affects about 1.5% of the world population and occurs more frequently in women than in men (2.5:1) [2,3]. Although the immune response plays an important role in RA, the aetiology is unknown. There are hypotheses which suggest that bacterial agents play an important role in the onset of the disease, but their causative link with RA remains controversial, because the studies have not established a strong enough association [4-6].

Mycoplasmas are a major cause of acute and chronic arthritis in animals and can induce arthritis in animal experimental models [7-9]. Mycoplasmas have been considered possible arthritogenic agents for humans since the 1960's when mycoplasmas were isolated from arthritic joints of animals, especially *Mycoplasma fermentans*, which was isolated from synovial fluids (SF) [10]. There is increasing evidence to suggest that mycoplasmas may play a role in RA [11-13]. The other mycoplasmas that are less frequently involved in human RA are: *M. pneumoniae, M. hominis, M. genitalium, M. salivarium, M. orale*, and *Ureaplasma urealyticum *[13]. The purpose of this study was to investigate *M. fermentans *in the bloodstream of patients with RA.

## Methods

### Subjects

One hundred and fifty two patients who attended the Rheumatology Service of the Hospital Manuel Avila Camacho del Instituto Mexicano del Seguro Social in Puebla, México were included in the study. A rheumatologist examined the patients and all fulfilled the American College of Rheumatology criteria. The patients' ages ranged between 25 and 79 yr. All patients with RA were in the acute phase of the disease and had not been under antibiotic treatments for at least six weeks before the sample was taken.

Sixty-seven individuals without RA, systemic lupus erythematosus (SLE), antiphospholipid syndrome (APS) or infectious disease were included in the study as healthy individuals, since in several cases of these diseases an inflammatory response in the joint is observed. Ages in the healthy individuals ranged between 20 and 60 yr. All healthy individuals were not under antibiotic or other drugs treatment. The ethics committee of the Hospital Manuel Avila Camacho del Instituto Mexicano del Seguro Social approved this study and informed patient consent was obtained.

### Specimens

Peripheral whole blood samples from patients and healthy individuals were collected in order to detect mycoplasmas by culture and direct PCR. Antibodies specific to *M. fermentans *were also investigated. Blood samples, which were collected in citrate-containing or non anticoagulant tubes, were stored at -20°C until use.

### Mycoplasmal culture

One hundred microliters of plasma of patients and healthy individuals were dip-inoculated in 900 μL of three different media: SP4 medium with glucose, SP4 medium with urea and SP4 medium with arginine, in order to isolate fermentative mycoplasmas, *U. Urealyticum *and *M. hominis *respectively. An SP4 tube with each media was incubated as control. Three serial ten-fold dilutions were incubated at 37°C until the indicator phenol red changed to yellow for fermentative mycoplasmas or red to non-fermentative, which indicate mycoplasma growth, on the other hand, cultures were considered negative when the indicator did not change after 30 days for *M. pneumoniae *and *M. fermentans*, 4 days for *M. hominis *and *U. urealyticum*. Control cultures were prepared as follows: one hundred microliters of *M. fermentans *PG18, *M. pneumoniae *Eaton strain, *M. hominis, M. penetrans *GTU and *U. urealyticum *were dip-inoculated into 900 μL of plasma, and three serial ten-fold dilutions were done into SP4 media with glucose, urea or arginine and incubated at 37°C for 48 hours or until the indicator changed showing mycoplasma growth. Likewise, one hundred microliters of each reference strains were dip-inoculated into 900 μL of the SP4 media with the respective substrate. Dilutions are recommended when tissues or corporal fluids are cultivated, because some inhibitory substances for mycoplasmas may be present. Positive mycoplasma cultures were confirmed using the PCR technique. All the blood samples were directly cultured on blood agar plates to detect any aerobes or aerobic facultative bacteria.

### DNA extraction from blood samples

Red blood cells (2.5 ml) were lysed with nanopure water (10 ml) at room temperature for 10 min and centrifuged at 3,000 × *g *for 10 min. The pellet containing white cells was treated as follow: Triton X-100 (1%, 10 ml) was added and mixed gently for 5 min. After centrifugation at 3,000 × *g *for 10 min, SDS (1%, 10 ml) was added and mixed gently for 10 min, NaCl (0.1 M, 10 ml) was added for 10 min followed by the addition of 10 ml of cold ethanol. After centrifugation at 10,000 × *g *for 10 min, the supernatant was discarded and the DNA was recovered in 500 μL of nanopure water and stored at -20°C. DNA was also extracted from a 1.4 ml whole blood sample seeded with 100 μL of *M. fermentans *PG18 for use as a positive control sample.

### DNA extraction from mycoplasmal cultures

DNA was extracted from Mycoplasma-positive cultures as follows: 1.5 ml of culture was centrifuged at 12,000 × *g *for 20 min, the supernatant was discarded and the pellet was suspended in 100 μL of a solution containing 10 mM Tris-HCl pH 8.3, 100 mM KCl, 2.5 mM MgCl_2_, 1% Tween-20, 1% Triton X-100 and 0.5 mg/ml of proteinase K. Samples were heated at 60°C for 60 min and immediately brought to 90°C for 10 min, then placed in an ice bath for 20 min and stored at -20°C [14].

### PCR assays

PCR detection of *Mycoplasma sp*. DNA using 16 s RNA gene primers AR1 and AR2 was performed according to Shidu *et al*. [14]. The primers used to identify species are shown in Additional file 1[15-19]. Amplification was performed in a total volume of 50 μL containing 50 mM KCl, 1.5 mM MgCl_2_, 10 mM Tri-HCl (pH 8.3), 0.2 mM of each dNTP (Amersham Pharmacia Biotech, USA) and 1 unit of ampliTaq polymerase (Gibco, BRL, USA). The sample to be analysed was always added last. A diluted lysate of *M. fermentans *PG18 or *M. pneumoniae *(Eaton strain) or *M. penetrans *GTU-54 or *M. hominis *or *U. urealyticum *corresponding to 100 colour-changing units and sterile water were used as positive and negative controls, respectively. Amplification consisted of a 10-min thermal delay step at 95°C, followed by 40 cycles comprising a 20 s denaturation step at 95°C, a 1-min annealing step at 62°C, a 1-min elongation step at 72°C, and a final 4-min elongation step at 72°C PCRS were performed with an automated thermocycler (MJ Research, Watertown, MA.). PCR products were analysed by 2% agarose gels electrophoresis and ethidium bromide staining [14].

### Antibody-specific ELISA assay

The antibodies IgM and IgG to *M. fermentans *PG18 in sera were measured by ELISA using *M. fermentans *PG18 whole cells adjusted to an OD 600 = 1 and performed as described by Horowitz *et al*., 1995 [20]. Samples with absorbance equal to or higher than the mean of the control group plus three standard deviations for IgM and IgG were considered positive.

### Antibody-specific immunoblotting assay

**Figure 1:**
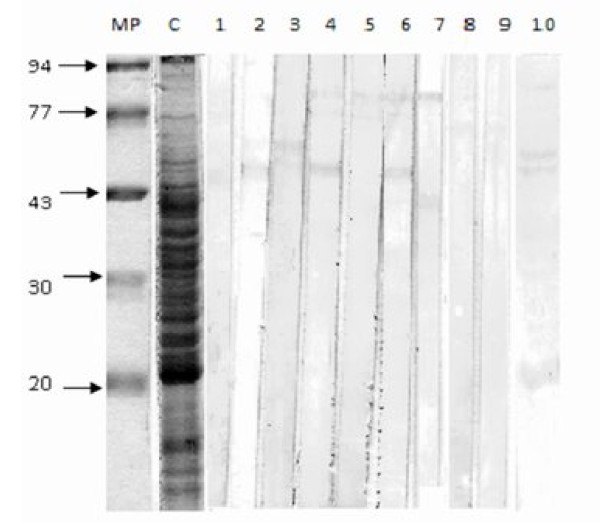
**Antibodies-specific to P70 and P48 were detected**. Line MW molecular weights; line 1 *M. fermentans *PG18 antigens extracted by heat; line 2, 4, 6, 8 IgM detection; line 3, 5, 7 IgG detection; line 9, 10 negative serum from healthy individuals; line 11 positive serum from an immunized rabbit with *M. fermentans *PG18.

The antigen used was *M. fermentans *PG18 whole cells adjusted to an OD 600 = 1 and 750 μL of this solution was mixed with 250 μL of sample buffer 4×(40% glycerol, 240 mM Tris/HCl pH 6.8, 0.04% bromophenol blue, 5% beta-mercaptoethanol) and boiled during ten minutes. Immunoblotting according to Horowitz *et al*. 1998 [21] was applied to detect IgM and IgG specific to *M. fermentans *PG18. The antibodies were detected by incubating 2 nitrocellulose (NC) strips in each patient's serum diluted 1:100 in PBS at 37°C for 2 h. Peroxidase-conjugates anti-human IgM or IgG were diluted 1:5000 in PBS and one strip was used in order to detect every one of the antibody isotypes. A rabbit was immunized with *M. fermentans *PG18 and the serum was used as positive control (line 11, figure 1). Peroxidase substrate was prepared according to manufacturer (Sigma Co.) and the bound peroxidase activity was visualized using the incubation of the NC strips for 5 to 20 min at room temperature. The enzymatic reaction was stopped and stabilized by washing the NC strips in a solution containing 13 ml of dioctyl sodium sulfosuccinate-ethanol in 37 ml of water.

### Statistical analysis

Chi squared test was done in order to compare the presence of antibodies against *M. fermentans *in patients and healthy individuals.

## Results

### Patients

Eighty seven patients with RA, 50 patients with SLE and 15 patients with APS were included in the study; all of them had varying times of disease, duration ranging from 2–25 years. All patients with autoimmune diseases were receiving weekly immunosuppressive therapy (50% receiving prednisone at 5–10 mg and/or methotrexate at 5–10 mg). All patients were receiving therapy with non-steroidal, anti-inflammatory drugs. Thirty percent of the patients had a history of recurrent respiratory or genitourinary tract infections, but at the time of specimen collection there were no signs of infectious disease.

### Mycoplasmal culture

All of the blood samples from patients with SLE, APS and healthy individuals were negative for mycoplasma by culture. Among the 87 peripheral blood samples from patients with RA, thirteen were considered culture-positive for *M. fermentans *because they changed the colour indicator of the media and the pure culture was then identified by PCR (Additional file 2).

### Detection of Mycoplasma DNA by Direct PCR

AR1 and AR2 genus-specific primers, which amplify a 16 s RNA gene fragment, were used to detect mycoplasmas in peripheral blood samples. *Mycoplasma *spp sequence was amplified in 13/87 samples from patients with RA and 5/50 samples from patients with SLE. All the peripheral blood samples from APS patients and healthy individuals were negative. Species-specific PCR primers were then used to determine which mycoplasmas were present. All samples from RA patients in which *Mycoplasma *spp DNA was amplified were positive for *M. fermentans *DNA, in blood samples from patients with SLE were detected by direct PCR to *M. fermentans *in 2/50 (2%), *M. hominis *in 2/50 (2%) and *U. urealyticum *in 1/50 (0.5%). Respect to the identification of *M. fermentans *in patients with RA, there was only concordance between culture and direct PCR in 6 patients, so we identified *M. fermentans *in 20/87 (23%) patients with RA (Additional file 2).

### Antibody-specific ELISA assay

IgM and IgG against *M. fermentans *PG18 were measured by ELISA. A threshold level above which samples were deemed positive was determined using a cut-off value generated from results from the control population. IgM against *M. fermentans *PG18 was detected in sera from 40/87 patients with RA and in sera of 7/67 healthy individuals. X^2 ^was calculated and association between the presence of IgM antibodies against *M. fermentans *PG18 and RA patients (p < .01). IgG was detected in sera from 48/87 RA patients and in sera from 7/67 healthy individuals. There was association between the absence of antibodies against *M. fermentans *PG18 and healthy individuals (p < 0.1) using X^2^. A higher percentage of patients than healthy individuals had antibodies against *M. fermentans *PG18 (Additional file 3).

### Antibody-specific immunoblotting assay

IgM-specific to *M. fermentans *PG18 was detected in sera from 35/87 patients with RA and in sera from 4/67 healthy individuals. IgG was detected in sera from 34/87 patients and in sera from 5/67 healthy individuals. Figure 1 shows representative results of immunobloting assay, IgM and IgG were detected to P70 and p48. There was a significant difference. X^2 ^was calculated (p < 0.1) (Additional file 3).

## Discussion

Infectious agents such as viruses, bacteria, and bacterial components have been hypothesized to play some role in the pathology of RA and other autoimmune diseases based on clinical and pathological findings in humans and animals [22-24]. However, this hypothesis remains unproven. Hernández *et al*. (1998) studied the recurrence and severity of infections in Mexican patients with RA and found that 48.7% of the patients suffered from infections related to treatment with steroids and methotrexate [25]. In this study we examined blood samples from RA, SLE, and APS patients for aerobic, aerobic facultative bacteria and mycoplasmas in particular, through culture, PCR, and immunological assays. Although all samples were negative for aerobic or aerobic facultative bacteria, 30% of the patients had a history of recurrent upper respiratory and genitourinary tract infections, 50% were being treated with prednisone and/or MTX, and 100% were being treated with nonsteroidal antinflammatory drugs. These findings are consistent with a role for infectious agents in RA. Mycoplasmas are putative agents in RA because they can produce arthritis in animals [26] and have also been detected in patients with RA [27] and other inflammatory arthritides [28]. However, results have been poorly reproduced, in part because of the difficulties of working with mycoplasmas. Since mycoplasmas are fastidious bacteria that are difficult to culture and identify and also may suffer phenotypic changes in culture, culture techniques and serological assays are not reliable diagnostic tests for mycoplasmal infection. Serology provides evidence of host contact with mycoplasmas, but does not provide evidence of the bacteria in the host. PCR techniques allow sensitive and specific detection of mycoplasmal DNA in various biological samples [29,30], but gives no information about the viable bacteria. To detect whether there is a causal relationship of microorganisms with arthritis it is important the viable bacteria as well as the immune response to the bacteria. That is why we used several techniques to diagnose mycoplasmal infection. In order to obtain reproducible results, we used standard techniques reported for isolation and PCR identification of mycoplasmas and all the risk factors of contamination were avoided.

The AR1 and AR2 primers have been reported as specific to the *Mycoplasma *genus (they do not cross-react with other bacteria) and can amplify 16 s RNA gene nucleotide sequences of 30 different species [14]. Using these primers and species-specific primers, we detected *M. fermentans *in 23% of patients with RA and in 2% of patients with SLE, but did not detect it in patients with APS or in healthy individuals.

*M. fermentans *has been previously detected in patients with RA and inflammatory diseases. Schaeverbeke *et al*. (1997) detected *M. fermentans *in two patients with RA and in one patient with a different inflammatory arthritis. They also reported the isolation of *M. hominis, M. salivarium, M. orale*, and *U. urealyticum *from SF and did not find concordance between culture and PCR [13]. Horowitz *et al*. (2000) detected *M. fermentans *DNA in SF from 17.6% of patients with RA and in one patient with undefined arthritis, but it was not detected by culture in any of these patients and they did not detect *M. fermentans *in other inflammatory or non-inflammatory arthropathies [31]. Gilroy *et al*. (2001) detected *M. fermentans *DNA in the SF of 17% of patients with RA and in 21% of patients with seronegative arthritis, and they did not detect it in patients with other arthropathies [11]. In contrast, Johnson *et al*. (2000) and Hoffman *et al*. (1997) reported higher percentages of detection of DNA of *M. fermentans *by PCR. The former reported *M. fermentans *in 31/34 patients with RA, and in 9/11 patients with non-rheumatoid inflammatory arthritis, and it was not isolated in patients with osteoarthritis. The latter did not detect *M. fermentans *in SF or tissues and did not use culture procedures [12,32]. Haier *et al*. (1999) identified *M. fermentans*, *M. pneumoniae*, *M. hominis *and *M. penetrans *by PCR in leucocytes from patients with RA [27]. In contrast, in this study we did not detect *M. pneumoniae *or *M. penetrans*. *M. hominis *and *U. urealyticum *were only identified by PCR in patients with SLE. Johnson *et al*. (2007) found by a very sensitive PCR a conserved mycoplasmal 16 S RNA of *M. pneumoniae *in the SF from patients with RA, osteoarthritis and nonrheumatoid inflammatory arthritis and it was not found in the SF from people with knee injuries or undergoing surgery for knee replacement [33].

Immune and inflammatory responses can be triggered or exacerbated by many factors including infectious agents. Aerobic and anaerobic bacteria, as well as viruses and mycoplasmas, have been considered as important agents in RA. Mycoplasmas have the ability to induce a broad range of inflammatory and immunological events. They are able to induce cytokine production and also activate macrophages, B and T lymphocytes. In this study we investigated whether antibodies against *M. fermentans *PG18 were present in sera from patients with RA and healthy individuals. Our results are similar to those reported by Horowitz *et al*. (2000), who detected antibodies against *M. fermentans *in 50% of patients with RA and 20% of patients with other arthritis; however there are differences respect to *M. fermentans *peptides detected, while Horowitz *et al*. detected antibodies to p107, p48 and p29, we reported antibodies to p70 and p48, the differences probably are due to the different strains and the technique used for the antigen extraction [31]. In contrast Hoffman *et al*. (1997) found antibodies against *M. hominis *in 55% of patients with RA and 88% juvenile RA patients and they also detect antibodies against this microorganism in patients with other inflammatory disease and controls [32]. Ramírez *et al*. (2005) performed a case-control study in which they show a statistical difference when they compare the presence of antibodies against *M. pneumoniae *in patients with RA and controls [34]. In our study, 20 out of 87 RA patients showed evidence of infection with *M. fermentans*. Thirteen patients had persistent infection with *M. fermentans *as evidenced by cultivable bacteria in the blood. All but two of these thirteen displayed an antibody response to *M. fermentans*. These results suggest that viable *M. fermentans *in blood of patients with RA is common, even though the role of bacteria is not known. In the other hand, it will be important to do studies in order to detect antibodies against *M. fermentans *glycolipid-antigen in patients with RA and other inflammatory arthritides, since Kawahito *et al*. (2008) detected to GGPL-III in 38.1% of RA patient's tissues [35].

There is a hypothesis that bacteria may trigger arthritis, and two models are often considered. One model postulates that arthritis is promoted by inflammation due to a chronic infection characterized by the long term presence of low levels of bacteria. The other postulates a "hit and run" phenomenon in which bacteria infect a joint for only a short period of time before being cleared [36], but initiate a cascade of inflammatory events while there, both nonspecific and specific responses that lead to inflammation and arthritis [37]. *M. fermentans*, which is frequently isolated from joints, may fit into the "hit and run" class. The main contribution of this study is that *M. fermentans *bacteremia was present in a significant number of patients with RA, supporting the idea that *M. fermentans *is a possible agent to consider in the pathology of RA. These results, and those of previous studies that also detected *M. fermentans *in RA patients, suggest that patients with RA have been exposed to *M. fermentans*, and that a high percentage of patients develop a systemic mycoplasmal infection[13,27,31], and the contradictory results on his isolation or identification are in part, because this mycoplasma may not be permanently in SF or tissues due to passive carriage or ubiquitous[31], since it has been found in pharynx, saliva peripheral mononuclear cells[38], another fact that support that *M. fermentans *can be associated with RA, was the study of Rivera *et al*. in an animal model, in which they showed that when this microorganism is inoculated in the trachea it can reach the joint [9]. The role of *M. fermentans *in initiating or perpetuating synovitis should be explored further, but three hypotheses can be considered with respect to the role of mycoplasmas in RA. The first proposes that *M. fermentans *is a cofactor of the disease in a genetically susceptible host and its role is in the induction of the autoimmune response. On the other hand, the second suggests that RA is produced by the reach of *M. fermentans *in the joint and the liberation of toxic substances or cytokines that produce joint damage [39]. With the same evidence, the third proposes that patients with RA are frequently under steroid treatment and steroids may favour systemic infections with *M. fermentans*.

Since RA has been associated with the presence of autoantibodies including rheumatoid factor, and the role of microorganisms is still unknown, the other important role in this disease is the genetic risk. Histocompatibility antigens (HLA-DR 1/4) in patients with RA have been studied, motif sequences like QKRAA, QRRAA. RRRAA or ESRRAL have been implicated in the susceptibility to develop RA. There are studies that determined the presence of share epitope in microorganisms[40]. Ebringer *et al*. (1992) show cross-reactivity between HLA-DR4 and *Proteus mirabilis *haemolysin [41] although in mycoplasmas there are not studies about the presence of shared epitope, it will be important to study this possibility.

## Conclusion

Our results are similar to those reported in the literature regarding the implication of *M. fermentans *in RA. The main contribution of our study is that only *M. fermentans *produce bacteremia in a higher percentage of patients with RA. IgM and IgG were detected against p70 and p48 *M. ferementans *antigens. These findings suggests that *M. fermentans *is common in these patients and it may enter through the respiratory tract and blood could be the medium to reach joints, where they may induce an inflammatory or immune response.

## Competing interests

The authors declare that they have no competing interests.

## Authors' contributions

CGJ carried out the samples collection, mycoplasmas' cultures and wrote the paper. JART carried out the PCR assays in order to identify mycoplasmas. DBR and SSS participated in the selection of the patients. EGL participated in the design and coordination. LC conceived the study, performed the statistical analysis and drafted the manuscript.

## Pre-publication history

The pre-publication history for this paper can be accessed here:



## Supplementary Material

Additional file 1 **Table 1**. Primers used in order to identify *Mycoplasma *species. Click here for file

Additional file 2 **Table 2**. Detection of *M. fermentans *by culture, direct PCR and IgM and IgG in patients with RA. Click here for file

Additional file 3 **Table 3**. Antibodies-specific ELISA and immunoblotting assays to *M. fermentans *in patients with RA and controls. Click here for file
