# Supplementary material for: Presence of Mycoplasma fermentans in the bloodstream of Mexican patients with rheumatoid arthritis and IgM and IgG antibodies against whole microorganism
Source: BMC Musculoskelet Disord. 2009 Aug 3;10:97. doi: 10.1186/1471-2474-10-97 (PMC2734754; doi:10.1186/1471-2474-10-97)
Supplement: Additional file 1 — Table 1. Primers used in order to identify Mycoplasma species. [file 1471-2474-10-97-S1.doc]

| Specie | Primer | Fragment amplified | Reference |
| --- | --- | --- | --- |
| *M. fermentans* | RW004 5´-GGACTATTGTCTAAACAATTTCCC–3’  RW005 5´-GGTTATTCGATTTCTAAATCGCCT-3´ | 206 bp | 15 |
| *M. pneumoniae* | MP5-1 5’-GAAGCTTATGGTACAGGTTGG-3’  MP5-2 5’-ATTACCATCCTTGTTGTAAGG-3’ | 144 bp | 16 |
| *M. penetrans* | MYCPENETP 5’-CATGCAAGTCGGACGAAGCA-3’ MYCPENETN 5’- AGCATTTCCTCTTCTTACAA-3’ | 407 bp | 17 |
| *U. urealyticum* | U4 5’-ACGACGTCCATAAGCAACT-3’  U5 5’-CAATCTGCTCGTGAAGTATTAC-3’ | 334 bp | 28 |
| *M. hominis* | RH1 5’-CAATGGCTAATGCCGGATACGC-3’  RH2 5’-GGTACCGTCAGTCTGCAAT-3’ | 429 bp | 19 |

Table 1. Primers used in order to identify *Mycoplasma* species.
