# Supplementary material for: Presence of Mycoplasma fermentans in the bloodstream of Mexican patients with rheumatoid arthritis and IgM and IgG antibodies against whole microorganism
Source: BMC Musculoskelet Disord. 2009 Aug 3;10:97. doi: 10.1186/1471-2474-10-97 (PMC2734754; doi:10.1186/1471-2474-10-97)
Supplement: Additional file 2 — Table 2. Detection of M. fermentans by culture, direct PCR and IgM and IgG in patients with RA. [file 1471-2474-10-97-S2.doc]

Table 2. Detection of *M. fermentans* by culture, direct PCR and IgM and IgG in patients with RA

| Patient | Culture* | Mycoplasma DNA Detection by Direct PCR** | ELISA | | Immunoblotting | |  | Patient | Culture* | Mycoplasma DNA Detection by Direct PCR** | ELISA | | Immunoblotting | |  | Patient | Culture* | Mycoplasma DNA Detection by Direct PCR** | ELISA | | Immunoblotting | |
| --- | --- | --- | --- | --- | --- | --- | --- | --- | --- | --- | --- | --- | --- | --- | --- | --- | --- | --- | --- | --- | --- | --- |
|  |  |  | IgM | IgG | IgM | IgG |  |  |  |  | IgM | IgG | IgM | IgG |  |  |  |  | IgM | IgG | IgM | IgG |
| 1 | - | + | + | + | + | + |  | 30 | - | - | - | - | - | - |  | 59 | - | - | - | - | - | - |
| 2 | - | + | - | - | + | + |  | 31 | - | - | - | + | - | - |  | 60 | - | - | - | - | - | - |
| 3 | + | - | + | + | + | + |  | 32 | - | - | + | + | + | - |  | 61 | - | - | + | + | - | - |
| 4 | + | + | + | + | + | + |  | 33 | - | - | - | - | - | - |  | 62 | - | - | + | + | - | - |
| 5 | + | + | + | + | + | + |  | 34 | - | - | - | - | - | - |  | 63 | - | - | - | - | - | - |
| 6 | + | + | + | - | - | + |  | 35 | - | - | + | + | + | + |  | 64 | - | - | - | - | - | - |
| 7 | - | + | + | + | + | + |  | 36 | - | - | + | + | + | + |  | 65 | - | - | - | + | - | - |
| 8 | + | - | + | + | + | + |  | 37 | - | - | + | + | + | + |  | 66 | - | - | - | - | - | - |
| 9 | + | + | + | + | - | - |  | 38 | - | - | + | + | + | + |  | 67 | - | - | + | + | - | - |
| 10 | - | + | + | + | + | + |  | 39 | - | - | - | - | - | - |  | 68 | - | - | + | + | - | - |
| 11 | + | - | + | + | - | + |  | 40 | - | - | - | - | - | - |  | 69 | - | - | - | - | - | - |
| 12 | + | - | - | - | - | - |  | 41 | - | - | - | - | - | - |  | 70 | - | - | - | - | - | - |
| 13 | + | + | + | + | + | + |  | 42 | - | - | - | - | - | - |  | 71 | - | - | - | + | - | - |
| 14 | + | - | + | + | + | - |  | 43 | - | - | + | + | + | + |  | 72 | - | - | - | - | - | - |
| 15 | + | + | + | + | + | + |  | 44 | - | - | + | + | + | + |  | 73 | - | - | - | + | - | - |
| 16 | + | - | + | - | + | + |  | 45 | - | - | - | - | - | - |  | 74 | - | - | - | - | - | - |
| 17 | + | - | - | - | - | - |  | 46 | - | - | - | - | - | - |  | 75 | - | - | - | - | - | - |
| 18 | - | + | + | + | + | + |  | 47 | - | - | + | + | - | - |  | 76 | - | - | + | + | + | - |
| 19 | - | + | + | + | + | + |  | 48 | - | - | - | - | - | - |  | 77 | - | - | - | - | - | - |
| 20 | - | + | + | + | + | + |  | 49 | - | - | - | - | - | - |  | 78 | - | - | + | + | + | + |
| 21 | - | - | - | + | - | - |  | 50 | - | - | - | - | - | - |  | 79 | - | - | + | + | + | + |
| 22 | - | - | + | + | + | + |  | 51 | - | - | + | + | + | + |  | 80 | - | - | - | - | - | - |
| 23 | - | - | - | - | - | - |  | 52 | - | - | + | + | + | + |  | 81 | - | - | - | + | + | + |
| 24 | - | - | + | + | + | + |  | 53 | - | - | - | - | - | - |  | 82 | - | - | - | - | - | - |
| 25 | - | - | + | + | + | + |  | 54 | - | - | - | - | - | - |  | 83 | - | - | + | + | + | + |
| 26 | - | - | - | - | - | - |  | 55 | - | - | - | + | - | - |  | 84 | - | - | + | + | + | + |
| 27 | - | - | + | + | + | - |  | 56 | - | - | - | + | - | - |  | 85 | - | - | - | - | - | - |
| 28 | - | - | - | + | - | - |  | 57 | - | - | - | - | - | - |  | 86 | - | - | - | - | - | - |
| 29 | - | - | - | - | - | - |  | 58 | - | - | - | - | - | - |  | 87 | - | - | - | + | + | + |

*Cultures were considered positive for *M. fermentans* when they changed the indicator of the media and the pure culture was identified by PCR.

** It was important to note that Mycoplasma DNA detection was done in blood samples before culture
