# Supplementary material for: Presence of Mycoplasma fermentans in the bloodstream of Mexican patients with rheumatoid arthritis and IgM and IgG antibodies against whole microorganism
Source: BMC Musculoskelet Disord. 2009 Aug 3;10:97. doi: 10.1186/1471-2474-10-97 (PMC2734754; doi:10.1186/1471-2474-10-97)
Supplement: Additional file 3 — Table 3. Antibodies-specific ELISA and immunoblotting assays to M. fermentans in patients with RA and controls. [file 1471-2474-10-97-S3.doc]

|  | ELISA | | Immunoblotting | |
| --- | --- | --- | --- | --- |
| n | IgM | IgG | IgM | IgG |
| RA Patients (87) | 40/87 (46%) | 48/87 (55%) | 35 (40%) | 34 (39%) |
| Controls (67) | 5/67 (7.5%) | 7/67 (10 %) | 4 (6%) | 5 (7.5%) |

Table 3. Antibodies-specific ELISA and immunoblotting assays to *M. fermentans* in patients with RA and controls.
